# Risk of intestinal and extra-intestinal cancers in patients with inflammatory bowel diseases: A population-based cohort study in northeastern Italy

**DOI:** 10.1371/journal.pone.0235142

**Published:** 2020-06-23

**Authors:** Martina Taborelli, Michele Sozzi, Stefania Del Zotto, Federica Toffolutti, Marcella Montico, Loris Zanier, Diego Serraino

**Affiliations:** 1 Cancer Epidemiology Unit, Centro di Riferimento Oncologico di Aviano (CRO) IRCCS, Aviano, Italy; 2 Friuli Venezia Giulia Cancer Registry, Centro di Riferimento Oncologico di Aviano (CRO) IRCCS, Aviano, Italy; 3 SC Data Warehouse, Flussi Informativi ed Epidemiologia, Agenzia Regionale di Coordinamento per la Salute, Udine, Italy; 4 Scientific Directorate, Centro di Riferimento Oncologico di Aviano (CRO) IRCCS, Aviano, Italy; National Health Research Institutes, TAIWAN

## Abstract

The cancer risk of patients with inflammatory bowel diseases (IBD) has not been well documented in southern Europe. This study aimed to evaluate the overall pattern of cancer risk among patients with IBD in Friuli Venezia Giulia, northeastern Italy. A population-based cohort study was performed through a record linkage between local healthcare databases and the cancer registry (1995–2013). We identified 3664 IBD patients aged 18–84 years, including 2358 with ulcerative colitis (UC) and 1306 with Crohn’s disease (CD). Sex- and age-standardized incidence ratios (SIRs) and 95% confidence intervals (CIs) were used to compare the cancer incidence of IBD patients with the general population. The cumulative cancer risk among IBD patients reached about 10% after 10 years of follow-up. A total of 246 cancers occurred among UC patients (SIR = 1.05, 95% CI: 0.92–1.19), and 141 among CD patients (SIR = 1.20, 95% CI: 1.01–1.41). As compared with the general population, no increased risk of colorectal cancers was observed for either UC or CD patients, whereas the risk of anal cancer was significantly elevated among UC patients (SIR = 6.03, 95% CI: 1.24–17.60). Increased risks were seen for specific extra-intestinal cancers, including corpus uteri (SIR = 2.67, 95% CI: 1.07–5.50) and kidney (SIR = 2.06, 95% CI: 1.03–3.69) among UC patients; thyroid (SIR = 5.58, 95% CI: 2.41–11.00) and skin non-melanoma (SIR = 1.86, 95% CI: 1.32–2.55) among CD patients. This population-based study showed that both UC and CD patients had a colorectal cancer risk similar to that of the general population. However, they were at a higher risk of developing certain extra-intestinal cancer types. Although detection biases cannot be excluded, the study findings pointed to a role of long-standing exposures to immunosuppressive therapies, underlying disease status, as well as the interactions with lifestyle factors. Our findings lent additional support to the need for monitoring the cancer burden in this at-risk population.

## Background

Inflammatory bowel diseases (IBD), consisting of ulcerative colitis (UC) and Crohn's disease (CD), are characterized by chronic inflammation of the gastrointestinal wall in genetically susceptible subjects who are exposed to environmental risk factors [1]. Epidemiological data have shown great variability in the IBD geographic distribution, with the highest incidence rates recorded in Western Countries, particularly in North America and Europe [2].

It is recognized that patients with IBD are at increased risk of developing intestinal cancers, as a result of chronic intestinal inflammation [3]. According to the most recent meta-analysis of population-based studies, the risk of developing colorectal cancers (CRC) in patients with both CD and UC was 1.7-fold higher than in the general population [4]. However, the latest population-based studies have shown either lack of CRC excess risks in IBD [5–9], or excess risks restricted to specific subgroup of patients [10–12]. Additionally to CRC, patients with IBD are also at increased risk of small bowel adenocarcinoma [5,13], ileo-anal pouch and rectal cuff cancer, and anal/perianal fistula cancers [7,14].

Elevated rates of various extra-intestinal tumors have also been associated with IBD [5,7–10,13,15–20]. Increased risks of lymphomas and cancers of the stomach, lung, breast, bladder, kidney, brain, non-melanoma skin, thyroid, and pancreas, have been documented in patients with CD [5,7–11,13,16,17,20,21]. Conversely, UC patients were shown to be at elevated risks of leukemia and hepatobiliary, breast, thyroid, non-melanoma skin, and prostate cancers [5,7–10,12,15,21,22].

Population-based studies of site-specific cancer risk in patients with IBD are still scarce. Although several investigations have been conducted in Canada [9,22], Asia [7,13] or, northern Europe [10,12,18], scanty evidence has emerged in southern European countries, including Italy. Accordingly, this study aimed to evaluate, in northeastern Italy, the overall pattern of cancer risk among patients with IBD, as compared to the corresponding general population.

## Materials and methods

### Data sources

A retrospective, population-based, cohort study was conducted using data from administrative healthcare databases of the Friuli Venezia Giulia region (1,200,000 inhabitants), north-eastern Italy. For the aims of this analysis, the following databases were used: 1) the regional health information system, which records patients’ personal data (e.g., sex, birth date, residence, vital status) and medical care history (e.g., hospital discharge, outpatient care, histopathological reports, medical exemptions, and drug prescriptions); and 2) the population-based cancer registry, which collects data on all new cancer cases occurring in the resident population of the Friuli Venezia Giulia region since 1995. Using a record linkage procedure, information derived from these databases were linked for the latest available period (i.e.,1995–2013). In observance of the Italian privacy laws, all the patients’ data were anonymous and linked by means of an anonymous unique identifier, randomly assigned to each patient. At time of first contact with the regional health system, all patients had signed an informed consent giving access to data in their medical records for research purposes. Furthermore, the Italian legislation identifies Cancer Registries as collectors of personal data for surveillance purposes without the need of explicit individual consent. Since neither direct nor indirect intervention on patients took place, the approval of a research ethics committee was not a requirement in this retrospective study.

### Study population

Patients were identified as IBD cases when they met one of the following conditions: first, at least one hospital discharge with an IBD-specific diagnostic code according to the International Classification of Disease, Ninth Edition (ICD-9) (i.e., ICD-9 codes: 555.x for CD or 556.x for UC); second, an exemption from medical charges because of IBD (regional codes: P28/1 for CD or P28/2 for UC); third, at least one IBD-related histopathological report derived from the ‘Diagnosis’ code of the Systematized Nomenclature of Medicine (SNOMED) II (i.e., code: D6216) or the SNOMED International Version 3.0 (i.e., codes: D541000, D541110, D541116, D541160). Thus, for each patient, the date of IBD diagnosis was defined as the first date when an IBD was registered under one of the three listed conditions.

For the purposes of this study, the analysis was restricted to all patients diagnosed with IBD during 1995–2013, while residing in the Friuli Venezia Giulia region, and aged 18–84 years at diagnosis. IBD patients with less than 5 years of residence in the Friuli Venezia Giulia region preceding IBD diagnosis, or a follow-up shorter than 90 days were excluded from the analyses. To avoid diagnostic ambiguities, 279 patients with codes for both CD and UC were also excluded. According to these criteria, the study population included 3664 IBD patients.

### Cancer classification

Cancer diagnoses were coded according to the International Classification of Diseases and Related Health Problems, 10th revision (ICD-10). Multiple primary cancers were included in site-specific analyses. The following cancer groups were analyzed: 1) all cancers; 2) all cancers, excluding non-melanoma skin; 3) intestinal cancers (i.e., small intestine, colorectal, and anal cancers, ICD-10 codes: C17-C21); and 4) extra-intestinal cancers (ICD-10 codes: C00-C16, C22-C97). Among extra-intestinal cancers, we considered the following subgroups: a) other digestive cancers (ICD-10 codes: C15-C16, C22-C26); b) respiratory tract cancers (ICD-10 codes: C30-C39); c) female genital organ cancers (ICD-10 codes: C51-C58); d) urinary tract cancers (ICD-10 codes: C64-C68); and e) hematological cancers (ICD-10 codes: C81-C96). In addition, we separately analyzed all cancer sites with at least four cases observed among all IBD patients.

### Statistical analysis

Person-years (PYs) at risk of cancer were computed from 90 days after the date of IBD diagnosis to the date of cancer diagnosis, to the date of death, to the date of the 85^th^ birthday, to the date of last follow-up, or to December 31, 2013, whichever came first. In the macro-group analyses, individuals were not censored at first cancer as they were still at risk of other types of cancers included. In the site-specific analyses, after a cancer diagnosis, patients did not further contribute follow-up time to the determination of PYs at risk for that specific cancer, but they continued to add follow-up time for other cancer sites. Moreover, patients with a history of cancer preceding their IBD diagnosis were not considered eligible to contribute to person-time at risk for that specific cancer site. For colon and rectal cancer-specific analyses, patients who had undergone surgery, in terms of intestinal resection, did not add person-time at risk for those cancers as appropriate.

Demographic and clinical characteristics of the cohort were presented as frequencies, and were compared through the chi-squared test between IBD type groups. To determine absolute risk estimates of incidence, the cumulative cancer incidence function over the follow-up period was estimated for each IBD type, treating death as a competing risk. Cumulative incidence differences between IBD type groups were assessed by means of the Gray's test.

To compare cancer incidence in IBD patients with the general population of the same age and sex, we estimated standardized incidence ratios (SIRs) as the ratio between observed and expected numbers of cancer cases. The expected number of cases was computed by multiplying the amount of PYs among IBD patients by sex and age specific cancer incidence rates in the general population of the Friuli Venezia Giulia region, yielded from the population-based cancer registry. Corresponding 95% confidence intervals (CIs) for SIRs were computed based on the assumption that the observed number of cases followed a Poisson distribution. All analyses were stratified by IBD type.

To identify potential risk factors associated with cancer development, hazard ratios (HRs) and 95% CIs were estimated using Fine and Gray’s regression models, accounting for death as a competing event. All models were adjusted for sex and age at IBD diagnosis (continuous variable).

The overall survival probabilities were estimated by means of the Kaplan-Meier method, and the log-rank test was used to compare survival rates.

All statistical analyses were performed using SAS (SAS Institute, Cary, NC, USA, version 9.4) and statistical significance was claimed for p<0.05 (two-tailed).

## Results

The 3664 IBD patients were followed-up for a total of 28,857 PYs of observation with a median follow-up time of 6.9 years (interquartile range, IQR: 2.8–12.8). We identified 2358 (64.4%) patients with UC (median age: 54 years), and 1306 (35.6%) with CD (median age: 51 years), who were followed-up for a median of 6.7 and of 7.1 years, respectively. The main characteristics of the cohort members are presented in Table 1.

**Table 1:** Distribution of 3664 patients with Inflammatory Bowel Disease (IBD) by selected characteristics and IBD type. Friuli Venezia Giulia, northeastern Italy, 1995–2013.

	All patients	IBD type		
		Ulcerative colitis	Crohn’s disease	
		(n = 2358)	(n = 1306)	
	No. (%)	No. (%)	No. (%)	*p*-value^a^
Sex				
Female	1753 (47.8)	1093 (46.4)	660 (50.5)	
Male	1911 (52.2)	1265 (53.6)	646 (49.5)	0.02
Age at IBD diagnosis (years)				
18–34	859 (23.4)	512 (21.7)	347 (26.6)	
35–54	1040 (28.4)	671 (28.5)	369 (28.2)	
55–69	856 (23.4)	556 (23.6)	300 (23.0)	
70–84	909 (24.8)	619 (26.2)	290 (22.2)	<0.01
Median (IQR)	53 (36–69)	54 (36–70)	51 (33–68)	
Calendar year at IBD diagnosis (years)				
1995–1998	885 (24.2)	535 (22.7)	350 (26.8)	
1999–2003	920 (25.1)	585 (24.8)	335 (25.6)	
2004–2008	899 (24.5)	603 (25.6)	296 (22.7)	
2009–2013	960 (26.2)	635 (26.9)	325 (24.9)	0.02
Primary sclerosing cholangitis				
No	3629 (99.0)	2335 (99.0)	1294 (99.1)	
Yes	35 (1.0)	23 (1.0)	12 (0.9)	0.87
Follow-up				
Median (IQR)	6.9 (2.8–12.8)	6.7 (2.8–12.3)	7.1 (2.9–13.5)	
Total person-years	28,857	18,299	10,558	

^a^Evaluated through χ^2^ test.

Abbreviations: IQR. Interquartile Range.

**Fig 1:**
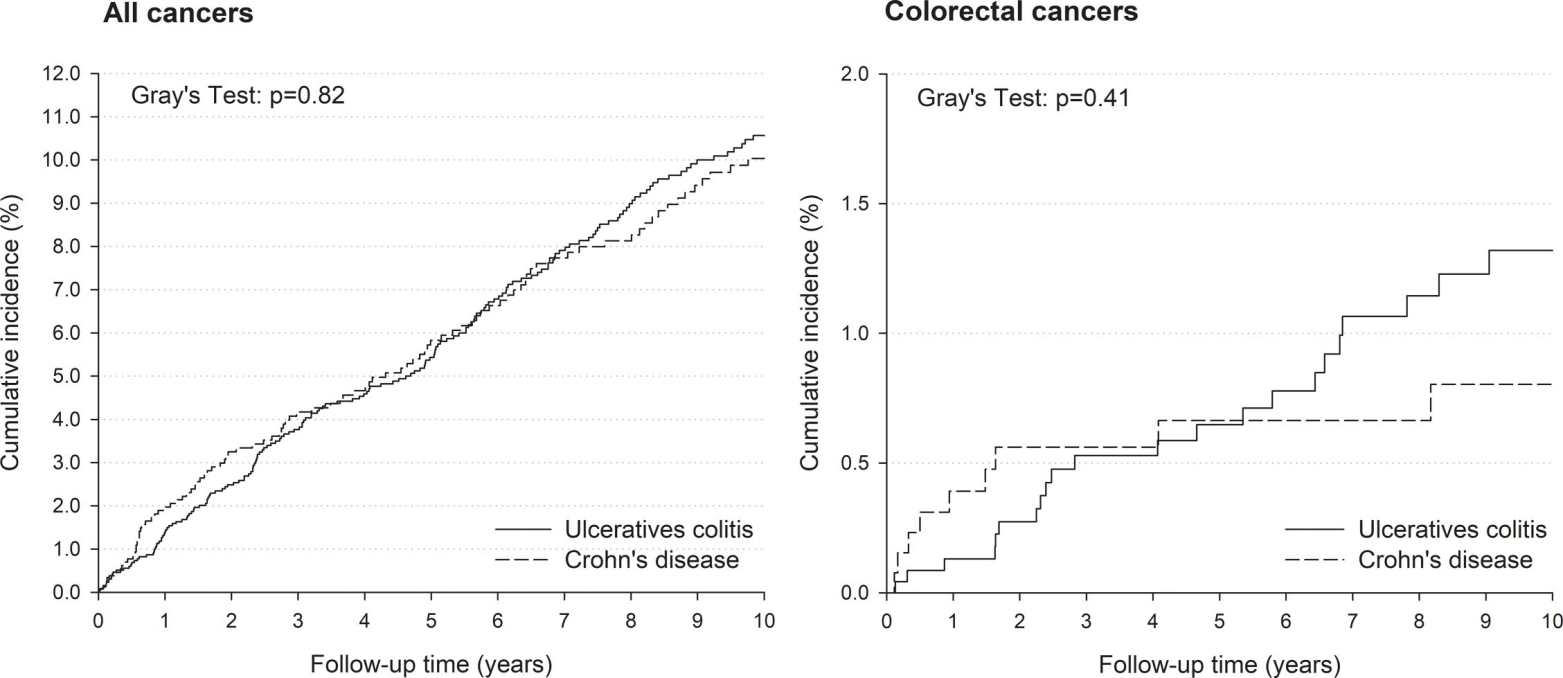
Cumulative incidence of all and colorectal cancers according to IBD type. Friuli Venezia Giulia, northeastern Italy, 1995–2013.

The cumulative risk of any cancer type increased steadily over the follow-up period. No differences emerged according to IBD type: the 5- and 10-year cumulative risks were 5.4% and 10.6% for UC patients, and 5.7% and 10.0% for CD patients, respectively (Fig 1). Similarly, the cumulative incidence of CRC rose over time but at a lower rate reaching 1.3% for UC and 0.8% for CD at 10 years.

Overall, 387 cancers were documented in 356 IBD patients during the observation period, resulting in a slight increase in cancer risk as compared with the general population (SIR = 1.10, 95% CI: 0.99–1.21) (Table 2). According to the IBD type, 246 cancers were diagnosed among 230 UC patients (SIR = 1.05, 95% CI: 0.92–1.19), whereas 141 were observed among 126 CD patients (SIR = 1.20, 95% CI: 1.01–1.41). No increased risk of CRC was observed for either CD or UC. However, among UC patients, the risk of anal cancer was significantly higher than in the general population (SIR = 6.03, 95% CI: 1.24–17.60). Non-melanoma skin cancer was the most frequent type in both IBD groups, though the effect was less evident in those with UC (SIR = 1.86, 95% CI: 1.32–2.55 in CD; SIR = 1.25, 0.94–1.65 in UC). Among patients with UC, statistically significant increased cancer risks were documented for corpus uteri (SIR = 2.67, 95% CI: 1.07–5.50) and kidney (SIR = 2.06, 95% CI: 1.03–3.69). Conversely, CD patients exhibited an increased SIR for thyroid cancer compared with the general population (SIR = 5.58, 95% CI: 2.41–11.00).

**Table 2:** Standardized Incidence Ratios (SIR) with corresponding 95% Confidence Intervals (CI) in patients with inflammatory bowel disease (IBD) for selected cancer sites, according to IBD type. Friuli Venezia Giulia, northeastern Italy, 1995–2013.

	Ulcerative colitis		Crohn’s disease		All patients
Cancer site (ICD-10 codes)	Obs./Exp.	SIR (95% CI)	Obs./Exp.	SIR (95% CI)	SIR (95% CI)
All	246/234.9	1.05 (0.92–1.19)	141/117.8	1.20 (1.01–1.41)	1.10 (0.99–1.21)
All but skin non-melanoma	194/191.8	1.01 (0.87–1.16)	103/96.1	1.07 (0.87–1.30)	1.03 (0.92–1.16)
*Intestinal (C17-C21)*^a^	33/26.0	1.27 (0.87–1.78)	12/12.8	0.93 (0.48–1.63)	1.16 (0.84–1.55)
Colon-rectum (C18-C20)	29/25.0	1.16 (0.78–1.67)	10/12.3	0.81 (0.39–1.50)	1.05 (0.74–1.43)
Colon (C18)	21/16.8	1.25 (0.78–1.92)	8/8.5	0.94 (0.40–1.85)	1.15 (0.77–1.65)
Rectum (C19-C20)	8/7.0	1.14 (0.49–2.24)	2/3.5	0.57 (0.07–2.05)	0.95 (0.45–1.74)
Anus (C21)	3/0.5	6.03 (1.24–17.60)	1/0.3	3.83 (0.10–21.40)	5.27 (1.44–13.50)
*Extra-intestinal (C00-C16*, *C22-C97*, *D09*.*0*,*D30*.*3*,*D41*.*4)*^b^	213/208.9	1.02 (0.89–1.17)	129/104.9	1.23 (1.03–1.46)	1.09 (0.98–1.21)
Oral cavity and pharynx (C00-C14)	5/5.9	0.85 (0.28–1.99)	0/2.9	0.00 (0.00–1.05)	0.57 (0.19–1.34)
Other digestive (C15-C16, C22-C26)^c^	22/27.8	0.79 (0.50–1.20)	14/13.5	1.03 (0.57–1.73)	0.87 (0.61–1.20)
Stomach (C16)	4/9.1	0.44 (0.12–1.13)	5/4.4	1.14 (0.37–2.66)	0.67 (0.31–1.27)
Liver (C22)	5/7.4	0.68 (0.22–1.58)	3/3.5	0.86 (0.18–2.51)	0.74 (0.32–1.45)
Pancreas (C25)	9/6.2	1.44 (0.66–2.74)	4/3.1	1.28 (0.35–3.27)	1.39 (0.74–2.37)
Respiratory tract (C30-C39)^d^	22/26.5	0.83 (0.52–1.26)	17/12.5	1.36 (0.79–2.18)	1.00 (0.71–1.37)
Larynx (C32)	4/3.1	1.30 (0.35–3.33)	2/1.4	1.39 (0.17–5.01)	1.33 (0.49–2.89)
Bronchus and lung (C34)	18/22.2	0.81 (0.48–1.28)	14/10.5	1.33 (0.73–2.24)	0.98 (0.67–1.38)
Melanoma (C43)	8/5.3	1.51 (0.65–2.97)	6/2.8	2.14 (0.79–4.67)	1.73 (0.94–2.90)
Skin non-melanoma (C44)	52/41.5	1.25 (0.94–1.65)	38/20.4	1.86 (1.32–2.55)	1.45 (1.17–1.79)
Breast (C50)	15/19.6	0.77 (0.43–1.26)	9/12.0	0.75 (0.34–1.42)	0.76 (0.49–1.13)
Female genital organs (C51-C58)^e^	13/6.8	1.90 (1.01–3.26)	5/4.2	1.19 (0.39–2.78)	1.63 (0.97–2.58)
Corpus uteri (C54)	7/2.6	2.67 (1.07–5.50)	0/1.6	0.00 (0.00–1.86)	1.65 (0.66–3.40)
Ovary (C56)	3/1.7	1.72 (0.36–5.03)	1/1.1	0.93 (0.02–5.16)	1.42 (0.39–3.63)
Prostate (C61)	25/25.6	0.97 (0.63–1.44)	13/11.8	1.10 (0.59–1.89)	1.02 (0.72–1.39)
Urinary tract (C64-C68, D09.0, D30.3, D41.4)^f^	29/20.0	1.45 (0.97–2.08)	7/9.4	0.74 (0.30–1.53)	1.22 (0.86–1.69)
Kidney (C64)	11/5.3	2.06 (1.03–3.69)	2/2.6	0.76 (0.09–2.76)	1.63 (0.87–2.79)
Bladder (C67, D09.0, D30.3, D41.4)	15/12.5	1.20 (0.67–1.98)	5/5.9	0.84 (0.27–1.96)	1.08 (0.66–1.68)
Thyroid gland (C73)	1/2.4	0.42 (0.01–2.31)	8/1.4	5.58 (2.41–11.00)	2.34 (1.07–4.45)
Hematological (C81-C96)^g^	11/12.9	0.86 (0.43–1.53)	7/6.6	1.07 (0.43–2.20)	0.93 (0.55–1.47)
Non-Hodgkin lymphoma (C82-C85, C96)	5/6.1	0.82 (0.27–1.92)	4/3.1	1.29 (0.35–3.30)	0.98 (0.45–1.86)
Multiple myeloma (C90)	4/2.4	1.68 (0.46–4.31)	2/1.2	1.68 (0.20–6.05)	1.68 (0.62–3.66)

^a^It includes small intestine (n = 2)

^b^It includes bone (n = 1), mesothelioma (n = 1), other connective and soft tissue (n = 2), brain (n = 3), other endocrine glands (n = 1), other and ill-defined sites (n = 7)

^c^It includes esophagus (n = 2), gallbladder (n = 1), and other and ill-defined digestive organs (n = 3)

^d^It includes heart (n = 1)

^e^It includes cervix uteri (n = 3), vulva (n = 2), and other and unspecified female genital organs (n = 2)

^f^It includes renal pelvis (n = 1) and other and unspecified urinary organs (n = 2)

^g^It includes leukemia (n = 3).

Abbreviations: obs, observed; exp, expected.

In both UC and CD patients, the risk of developing cancer of any type was higher for males (HR = 1.41, 95% CI: 1.08–1.84 in UC; HR = 1.51, 95% CI: 1.05–2.17 in CD) as compared to females, and for those older than 34 years at IBD diagnosis (HR = 8.60, 95% CI: 4.55–16.22 and HR = 8.47, 95% CI: 4.07–17.60 for UC and CD aged ≥70 years, respectively) vs IBD patients aged 18–34 years at diagnosis (Table 3). Risk estimates for CRC showed a direct association with sex among UC patients only (HR = 2.26, 95% CI: 1.00–5.11 for males vs females) (Table 3). As expected, primary sclerosing cholangitis was associated with an increased risk of CRC among patients with UC (HR = 14.09, 95% CI: 4.72–42.40).

**Table 3:** Hazard Ratios (HRs)^a^ for all and colorectal cancers and corresponding 95% Confidence Intervals (CIs) among patients with Inflammatory Bowel Disease (IBD) according to selected variables and IBD type. Friuli Venezia Giulia, northeastern Italy, 1995–2013.

	All cancers				Colorectal cancers			
	Ulcerative colitis		Crohn’s disease		Ulcerative colitis		Crohn’s disease	
	% cases	HR (95% CI)	% cases	HR (95% CI)	% cases	HR (95% CI)	% cases	HR (95% CI)
Sex								
Female	8.2	1^b^	8.3	1^b^	0.7	1^b^	1.1	1^b^
Male	11.1	1.41 (1.08–1.84)	11.0	1.51 (1.05–2.17)	1.7	2.26 (1.00–5.11)	0.5	0.55 (0.14–2.10)
Age at IBD diagnosis (years)								
18–34	2.2	1^b^	2.6	1^b^	0.8	1^b^	0.0	-
35–54	7.9	3.84 (2.01–7.33)	7.3	3.01 (1.42–6.37)	0.9	1.11 (0.31–3.93)	0.5	1^b^
55–69	17.6	9.61 (5.17–17.89)	16.7	7.29 (3.61–14.73)	2.3	3.11 (1.02–9.50)	0.7	2.47 (0.35–17.20)
≥70	11.0	8.60 (4.55–16.22)	13.8	8.47 (4.07–17.60)	1.0	1.97 (0.57–6.76)	2.1	8.05 (1.83–35.38)
Calendar year at IBD diagnosis (years)								
1995–1998	15.7	1^b^	15.1	1^b^	1.9	1^b^	1.4	1^b^
1999–2003	12.5	1.01 (0.74–1.38)	10.8	0.84 (0.55–1.28)	1.7	1.35 (0.56–3.28)	0.6	0.41 (0.07–2.31)
2004–2008	8.1	0.82 (0.57–1.18)	9.8	1.13 (0.72–1.78)	1.0	1.00 (0.36–2.77)	1.0	0.78 (0.18–3.41)
2009–2013	3.8	0.85 (0.53–1.37)	2.5	0.51 (0.24–1.09)	0.5	1.10 (0.32–3.78)	0.0	-
Primary sclerosing cholangitis								
No	9.7	1^b^	9.7		1.1	1^b^	0.8	
Yes	13.0	1.36 (0.47–3.90)	0.0	-	13.0	14.09 (4.72–42.40)	0.0	-

^a^Estimated using Fine-Gray models adjusted for sex and age at IBD diagnosis

^b^Reference category.

**Fig 2:**
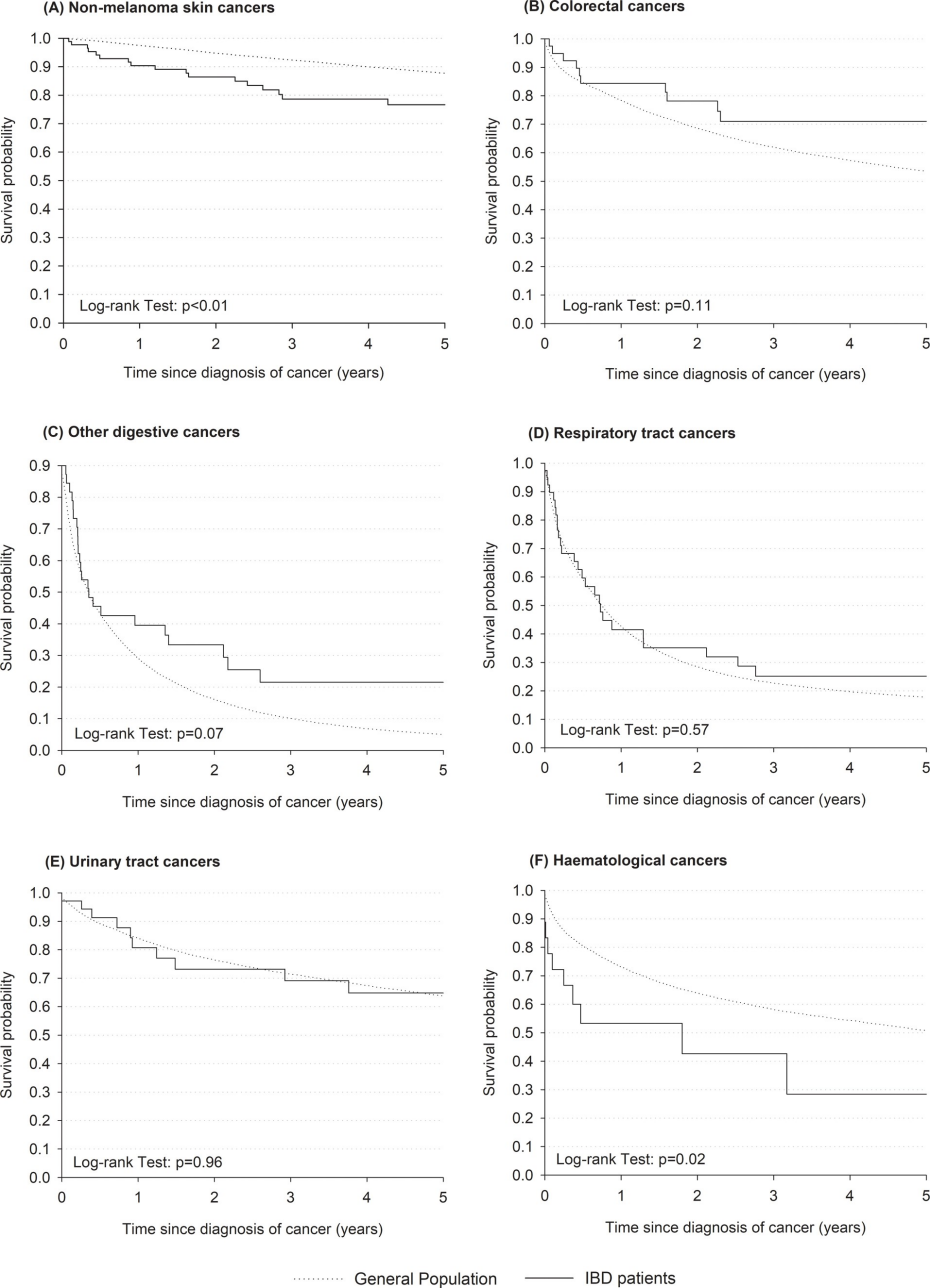
Kaplan-Meier estimates of survival probabilities following diagnosis of non-melanoma skin (A), colorectal (B), other digestive (C), respiratory tract (D), urinary tract (E), or haematological (F) cancers in patients with inflammatory bowel disease (IBD) and in the general population of Friuli Venezia Giulia region. Friuli Venezia Giulia, northeastern Italy, 1995–2013.

IBD patients who developed non-melanoma skin cancers showed a lower 5-year survival probability than the general population with the same tumor (77% vs 88%, log-rank test p<0.01) (Fig 2). Also those with hematological cancers had a worse prognosis compared to the general population, with 5-year survival rate particularly low at 28% vs 51% (log-rank test p = 0.02). No differences emerged for colorectal, extra-intestinal digestive, respiratory tract, and urinary tract cancers.

## Discussion

The results of this population-based cohort study indicate that the cumulative risk of cancer among IBD patients reached about 10% after 10 years of follow-up, corresponding to a slight increase in cancer risk compared to the background population. The CRC risk among both UC and CD patients was similar to that expected in the general population, whereas among UC patients the risk of anal cancer was significantly higher. Increased cancer risks were seen also for certain extra-intestinal cancers, including corpus uteri and kidney among UC patients; and thyroid and skin non-melanoma among CD patients.

The lack of excess CRC risk is consistent with findings from several recent population-based studies, showing a low or absent risk of CRC in both UC and CD patients [5–9,11,18]. Although a declining risk with respect to earlier investigations has been shown in most studies published since 2000, other current findings still show a two-fold increased risk [4,9,24]. Notably, a recent, large population-based cohort study with about 50 years of follow-up has found that individuals with UC were at increased risk of developing CRC but the excess risk decreased over time [12]. The lower than expected CRC incidence in our study population could be explained by several factors known to influence the risk of IBD-related CRC, including genetics, diet, chemoprevention, and differences in colonoscopy surveillance [25]. In addition, the duration of follow-up in our study population (median: 7 years), might have been insufficient to detect any increase. In fact, it is known that the risk of CRC, which is driven by the duration of colitis, begins to increase 8–10 years after the diagnosis of IBD and increases over time [26]. Also the exclusion of 20 cases of CRC diagnosed within three months since the diagnosis of IBD might have led to an underestimation of the risk. Indeed, when we conducted a sensitivity analysis by reducing the exclusion time from three months to one month since IBD diagnosis, the risk of CRC was higher (SIR = 1.21, 95% CI: 0.88–1.61). Nevertheless, in line with other studies [18], we excluded early cancers to avoid: 1) increased detection of cancer after IBD diagnosis owing to increased health care contact/detection; 2) simultaneous diagnosis of IBD and cancer as a result of overlapping symptoms and diagnostic procedures; and 3) IBD/cancer misclassification.

Among patients with IBD, we found a two-fold higher risk of developing CRC in males than in females with UC, while no sex differences emerged among CD patients. These findings are in agreement with a large, population-based, cohort study conducted in Sweden, which showed that IBD males had a 60% higher risk of CRC than females even though the effect of gender was limited to patients with more than 10 years of follow-up and those aged less than 45 years at diagnosis [27]. The risk of CRC was also higher in UC patients affected by primary sclerosing cholangitis than in non-affected ones. Although primary sclerosing cholangitis is a well-known risk factor for CRC in UC patients, it is unclear whether PSC has the same influence in patients with CD. Indeed, the few studies conducted on this topic have proposed conflicting results [28].

Little is known about the link of IBD to the risk of anal cancer, a rare neoplasm. The significantly higher risk of anal cancers found among UC patients was in contrast with previous studies that reported an association with CD only [7,29,30]. However, our results should be interpreted with caution since they are based on a low number of anal cancer cases (n = 3 and n = 1 among UC and CD, respectively).

It is known that IBD patients are at increased risk of extra-intestinal cancers [29], with the extent of risk depending on cancer type and IBD type [7,9–11,13,18,22,31]. Immunosuppressive therapies may play a key role in the development of extra-intestinal cancers by impairing immunosurveillance of cancer cells or inducing DNA damage [32]. A high risk of developing lymphomas and non-melanoma skin cancers associated with the use of thiopurine has been well described among IBD patients [29,33,34]. Our findings did not reveal an excess risk of lymphoproliferative malignancies. Nonetheless, patients with CD -and to some extent also patients with UC- were found to be at an increased risk of non-melanoma skin cancers compared with the general population, in line with previous studies [35]. To this regard, patients with IBD may be at increased risk of NMSC due to the immunosuppressive medications used to treat the disease and/or the underlying immune dysfunction of IBD. However, it is difficult to distinguish the role of IBD *per se* from the effect of immunosuppressant therapies. It is generally agreed that the risk of NMSC is related to drug-induced higher photosensitivity, and it increases with the duration of therapy [36].

We found that UC patients had an increased risk of corpus uteri cancer. Although some studies have suggested that female sex hormones may augment the risk of IBD, it is unclear whether an excess risk of female reproductive cancers exists in this population [18,37]. Several studies have failed to demonstrate a higher risk of female genital cancers among patients with IBD than in the general population [18,38]. Conversely, some authors have observed an increased risk of cervical cancer in IBD patients treated with immunosuppressants. To this regard, immunosuppressive therapies for patients with IBD seems to play an important role in facilitating the replication or persistence of the Human Papilloma Virus by compromising the local immunosurveillance [38]. We also observed an elevated risk of kidney cancer among UC patients. In most of the previous IBD population-based studies, no overall excess risk of urinary tract cancers, including kidney and bladder cancers, has been reported [22,31], apart from a Danish study that found a significantly increased risk of urinary tract cancers associated with the use of azathioprine [39].

The risk of thyroid cancer was increased among patients affected by CD. Although IBD, and in particular CD, has been associated with autoimmune thyroid disorders (e.g., Hashimoto’s thyroiditis and Grave’s disease), data on the association of IBD with thyroid cancer is scanty [40]. Our results are in line with those described in a US large investigation which have reported that CD, not UC, was associated with a high risk of thyroid cancer [20]. On the contrary, a recent meta-analysis reported that patients with UC had an increased risk of thyroid cancer, while patients with CD did not [23]. It is possible that the inflammatory response of IBD itself and the use of immunosuppressants may contribute to the development of thyroid cancer.

As for most investigations involving administrative healthcare databases, the main limit of this study is the lack of diagnostic validation. Nevertheless, we are confident that the combined use of three different sources of administrative databases of the Friuli Venezia Giulia region–which have served as sources for several published research articles [41–43]- have allowed reaching a large group of IBD patients with different types of access to health services. Indeed, hospital discharge records have been largely used in many studies for IBD case ascertainment [44–46] while inclusion of medical exemptions has been effectively used in previous studies on IBD or other chronic conditions [45,47]. Furthermore, pathological archives were included in a recent investigation [48].

Other limitations included the lack of information about patients’ lifestyle habits (e.g., smoking, sun exposure, and alcohol consumption), which are not regularly collected in the population-based administrative databases of the Friuli Venezia Giulia region. Furthermore, the study had a limited power to assess the excess risk of specific cancer sites, calling for caution in the interpretation of results. Finally, we could not rule out the possibility of detection bias as IBD patients could have had a higher rate of cancer detection than the general population due to their greater likelihood of seeking health care and needing more medical visits. To address this issue, we performed a sensitivity analysis computing SIRs according to time since IBD diagnosis (<1, 1–3, >3 years). In all patients, the risk of developing cancer of any type was substantially similar in all three periods. Subgroup analysis by IBD type showed that the risk was highest in the first year after IBD diagnosis among CD patients, while the effect was not evident among patients with UC (Table in S1 Table). Thus, detection bias seemed to only partially explain the highest risk of cancer in the first year following IBD diagnosis -particularly among CD patients- as well as the increased risk of less severe cancers such as NMSC. Furthermore, the lack of increased risk documented among UC patients in the first period pointed to pathways non totally attributable to detection biases.

Despite these limitations, the main strengths of this study were: i) its population-based design, which ensured a complete follow-up of the entire population of the Friuli Venezia Giulia region; ii) the high degree of completeness and accuracy of cancer incidence data, as they were collected by the population-based Friuli Venezia Giulia cancer registry; and iii) the size of the study, the largest population-based cohort in southern Europe and the first one in Italy–to the best of our knowledge- in which the association between IBD and specific cancers was investigated.

In conclusion, this population-based study showed that both UC and CD patients had a CRC risk similar to that of the general population. However, they were at a higher risk of developing certain extra-intestinal cancer types. Although detection biases cannot be excluded, the study findings pointed to a role of long-standing exposures to immunosuppressive therapies, underlying disease status, as well as the interactions with lifestyle factors. Our findings lent additional support to the need for monitoring the cancer burden in this at-risk population.

## Supporting information

S1 Dataset (XLS)

S1 Table Standardized Incidence Ratios (SIR) with corresponding 95% Confidence Intervals (CI) in patients with Inflammatory Bowel Disease (IBD) for selected cancer sites, according to time since IBD diagnosis (<1, 1–3, >3 years) and IBD type. Friuli Venezia Giulia, northeastern Italy, 1995–2013.
(DOCX)
