# Supplementary material for: Risk of intestinal and extra-intestinal cancers in patients with inflammatory bowel diseases: A population-based cohort study in northeastern Italy
Source: PLoS One. 2020 Jun 23;15(6):e0235142. doi: 10.1371/journal.pone.0235142 (PMC7310697; doi:10.1371/journal.pone.0235142)
Supplement: S1 Table — Friuli Venezia Giulia, northeastern Italy, 1995–2013. (DOCX) [file pone.0235142.s002.docx]

Table. Standardized incidence ratios (SIR) with corresponding 95% confidence intervals (CI) in patients with inflammatory bowel disease (IBD) for selected cancer sites, according to time since IBD diagnosis (<1, 1-3, >3 years) and IBD type. Friuli Venezia Giulia, northeastern Italy, 1995-2013.

|  | **Ulcerative colitis** | |  | **Crohn’s disease** | |  | **Total** |
| --- | --- | --- | --- | --- | --- | --- | --- |
| **Cancer type** | Obs./Exp. | SIR (95% CI) |  | Obs./Exp. | SIR (95% CI) |  | SIR (95% CI) |
|  |  |  |  |  |  |  |  |
| **All** |  |  |  |  |  |  |  |
| Time since IBD diagnosis (years) |  |  |  |  |  |  |  |
| <1 | 19/22.1 | 0.86 (0.52-1.35) |  | 22/10.3 | 2.14 (1.34-3.23) |  | 1.27 (0.91-1.72) |
| 1-3 | 60/50.3 | 1.19 (0.91-1.53) |  | 25/23.7 | 1.06 (0.68-1.56) |  | 1.15 (0.92-1.42) |
| >3 | 167/162.7 | 1.03 (0.88-1.19) |  | 94/83.9 | 1.12 (0.91-1.37) |  | 1.06 (0.93-1.19) |
| **All but skin non-melanoma** |  |  |  |  |  |  |  |
| Time since IBD diagnosis (years) |  |  |  |  |  |  |  |
| <1 | 16/18 | 0.89 (0.51-1.44) |  | 17/8.4 | 2.02 (1.18-3.24) |  | 1.25 (0.86-1.76) |
| 1-3 | 50/41.1 | 1.22 (0.90-1.60) |  | 18/19.3 | 0.93 (0.55-1.47) |  | 1.13 (0.87-1.43) |
| >3 | 128/132.9 | 0.96 (0.80-1.15) |  | 68/68.5 | 0.99 (0.77-1.26) |  | 0.97 (0.84-1.12) |
| **Intestinal** |  |  |  |  |  |  |  |
| Time since IBD diagnosis (years) |  |  |  |  |  |  |  |
| <1 | 3/2.5 | 1.21 (0.25-3.55) |  | 5/1.1 | 4.40 (1.43-10.30) |  | 2.22 (0.96-4.37) |
| 1-3 | 9/5.6 | 1.60 (0.73-3.04) |  | 3/2.6 | 1.15 (0.24-3.37) |  | 1.46 (0.75-2.55) |
| >3 | 21/18 | 1.17 (0.72-1.79) |  | 4/9.1 | 0.44 (0.12-1.12) |  | 0.92 (0.60-1.36) |
| **Extra-intestinal** |  |  |  |  |  |  |  |
| Time since IBD diagnosis (years) |  |  |  |  |  |  |  |
| <1 | 16/19.6 | 0.82 (0.47-1.33) |  | 17/9.2 | 1.86 (1.08-2.97) |  | 1.15 (0.79-1.61) |
| 1-3 | 51/44.7 | 1.14 (0.85-1.50) |  | 22/21.1 | 1.04 (0.65-1.58) |  | 1.11 (0.87-1.40) |
| >3 | 146/144.8 | 1.01 (0.85-1.19) |  | 90/74.8 | 1.20 (0.97-1.48) |  | 1.07 (0.94-1.22) |
| **Skin non-melanoma** |  |  |  |  |  |  |  |
| Time since IBD diagnosis (years) |  |  |  |  |  |  |  |
| <1 | 3/3.9 | 0.77 (0.16-2.26) |  | 5/1.8 | 2.74 (0.89-6.40) |  | 1.40 (0.60-2.76) |
| 1-3 | 10/8.9 | 1.13 (0.54-2.07) |  | 7/4.1 | 1.69 (0.68-3.48) |  | 1.31 (0.76-2.09) |
| >3 | 39/28.7 | 1.36 (0.97-1.86) |  | 26/14.5 | 1.80 (1.17-2.63) |  | 1.51 (1.16-1.92) |
|  |  |  |  |  |  |  |  |

Abbreviations: obs, observed; exp, expected.
